# Janzen-Connell effects shape gene flow patterns and realized fitness in the tropical dioecious tree *Spondias purpurea* (ANACARDIACEAE)

**DOI:** 10.1038/s41598-020-61394-4

**Published:** 2020-03-12

**Authors:** E. Jacob Cristóbal-Pérez, Eric J. Fuchs, Ulises Olivares-Pinto, Mauricio Quesada

**Affiliations:** 1 Instituto de Investigaciones en Ecosistemas y Sustentabilidad, Universidad Nacional Autónoma de México, Morelia, Michoacán México; 2 Laboratorio Nacional de Análisis y Síntesis Ecológica, Escuela Nacional de Estudios Superiores Unidad Morelia, Universidad Nacional Autónoma de México, Morelia, Michoacán México; 3 Escuela Nacional de Estudios Superiores Unidad Juriquilla, Universidad Nacional Autónoma de México, Santiago de Querétaro, Querétaro México; 4 Escuela de Biología, Universidad de Costa Rica, San José, 11501-2060 Costa Rica

**Keywords:** Molecular ecology, Ecological genetics

## Abstract

Pollination and seed dispersal patterns determine gene flow within plant populations. In tropical forests, a high proportion of trees are dioecious, insect pollinated and dispersed by vertebrates. Dispersal vectors and density dependent factors may modulate realized gene flow and influence the magnitude of Fine Scale Genetic Structure (FSGS), affecting individual fitness. *Spondias purpurea* is a vertebrate-dispersed, insect-pollinated dioecious tropical tree. We assessed the influence of sex ratio, effective and realized gene flow on genetic diversity, FSGS and individual fitness within a 30 ha plot in the tropical dry forest reserve of Chamela-Cuixmala, Mexico. All individuals within the plot were tagged, geo-referenced and sampled for genetic analysis. We measured dbh and monitored sex expression during two reproductive seasons for all individuals. We collected seeds directly from maternal trees for effective pollen dispersal analysis, and analyzed established seedlings to assess realized pollen and seed dispersal. Nine microsatellite loci were used to describe genetic diversity parameters, FSGS and gene flow patterns among different size classes. A total of 354 individuals were located and classified into three size classes based on their dbh (<10, 10–20, and >20 cm). Population sex ratios were male biased and diametric size distributions differed among sexes, these differences may be the result of precocious male reproduction at early stages. Autocorrelation analyses indicate low FSGS (Fj <0.07) across all size classes. Long realized pollen and seed dispersal and differences among effective and realized gene flow were detected. In our study site low FSGS is associated with high gene flow levels. Effective and realized gene flow indicate a population recruitment curve indicating Janzen-Connell effects and suggesting fitness advantages for long-distance pollen and seed dispersal events.

## Introduction

Dispersal is a central ecological mechanism that involves the movement of genes or individuals from one location to another. In plants, gene movement occurs through pollen flow (paternal gamete dispersal) and seed dispersal, which is facilitated by animals such as pollinators and seed dispersers or abiotic agents like wind and water^1,2^. Gene flow patterns are important for population and conservation genetics since they directly influence genetic diversity within populations and determine the genetic structure among populations^3^. Within plant populations, limited gene flow could result in fine spatial genetic structure (FSGS), that is, the non-random spatial distribution of genotypes^4^, resulting in neighborhoods of related individuals at a restricted spatial scale^4,5^. Other factors besides gene flow may generate FSGS, including colonization history^6,7^, demographic changes^8–10^ and the mating system and sexual expression of plants^3,11^.

Spatial distribution patterns in plants are determined by seed dispersal patterns and post-dispersal processes such as seed predation and seedling survival. For example, in zoochorous plants^12^ short-range dispersers forage near fruiting trees, resulting in higher seed deposition near maternal plants and FSGS. Further, seeds may accumulate in roosting or sleeping sites which would again result in aggregated dispersal and consequently resulting in FSGS^12^. According to the Janzen-Connell hypothesis, the establishment and survivorship of seedlings near maternal or conspecific plants could be affected by negative density-dependent processes such as intra-specific competition, host-specific predation, herbivory or pathogens^13,14^. Although density-dependence is commonly accepted as a factor responsible for high tropical diversity^15^, its influence on FSGS patterns is poorly understood. Proximate density dependent factors may increase mortality of seedlings near conspecifics or at high densities^13,14^, reducing FSGS. Dioecious trees are relatively common in tropical forests (ca. 27% trees)^16–18^. Dioecy is a reproductive system in which the male and female reproductive functions exist in separate individuals. A high proportion of dioecious species share reproductive traits such as small insect pollinated flowers and fleshy fruits dispersed by vertebrates^18,19^. Although dioecious trees are frequent in tropical forests, gene flow patterns^20,21^ and FSGS have been rarely studied^11,22^. In dioecious trees the magnitude of FSGS will depend, on the one hand, on the movement ability and behaviour of pollinators and seed dispersers and, on the other hand, on demographic traits associated with the reproductive system, such as sexual ratio and spatial distribution of adults. Theory of density-dependent animal pollination assumes that tree species occurring at low densities receive pollen from fewer individuals than trees in denser populations, and from longer pollen flow distances^23–26^. Theory predicts a 1:1 sex ratio maintained by negative frequency-dependent selection^27^, however intersex differences in growth, reproduction and survival may result in biases in sex ratio^28^. Specifically, males are expected to have higher growth rates, survivorship and reproduction than females, due to the higher energy costs associated with gamete and fruit production in females; resulting in male-biased sex ratios^29^. For dioecious plants, the spatial distribution of male and female trees as well as the population sex ratios could affect seed disperser behaviour playing an important role in determining FSGS patterns.

Fitness may be defined as an individual’s genetic contribution to future generations^30^. The number of surviving offspring of a given individual in the population, determined through paternity analyses, is a good proxy of plant fitness^31,32^. In plants, individual fitness will depend on their ability to produce viable seeds (i.e. fecundity) and the success of seeds to disperse and establish (i.e. survival). Therefore, factors affecting individual plant fitness as pollen gene flow distances, seed or seedling predation near maternal trees may affect FSGS formation. Due to the difficulty to estimate overall fitness in natural conditions, fitness analyses frequently rely on traits associated with one component of individual fitness, generally associated to fecundity (*e.g*. fruit production, seed production)^33^, ignoring the transition between seed formation and seedling emergence^34^. An approach to analyze fitness and fill the gap in this demographic transition is to estimate effective pollen flow and realized pollen and seed dispersal. Effective pollen dispersal is the pollen flow that occurs before seed dispersal, while realized pollen and seed dispersal refer to pollen and seed flow measured on established seedlings and juveniles^35^. This approach allows the evaluation of individual fitness through an estimate of the number of offspring by each parental plant, and secondly to analyse the contribution of gene flow patterns on FSGS.

In this study, we analyze fine-scale genetic structure (FSGS) in relation to gene flow patterns and fitness on the dioecious, insect pollinated and vertebrate seed dispersed tree, *Spondias purpurea* L. We analyse the effect of demography, gene flow and sex ratio on effective gene flow patterns. Specifically, we evaluate the following objectives: (1) Determine the relationship between population sex ratios, size classes and FSGS; (2) Determine the relationship between gene flow patterns via seed and pollen dispersal, with respect to FSGS, and (3) Determine the relationship of effective and realized pollen dispersal, as a proxy of plant fitness in relation to FSGS.

## Results

### Sexual ratios

**Figure 1:**
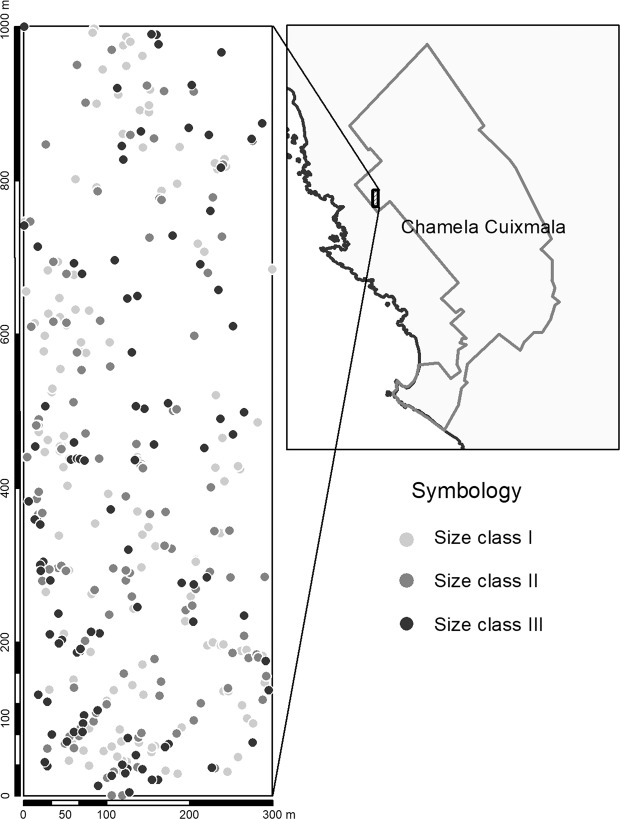
Spatial distribution Spondias purpurea individuals in a 30 ha plot located in Chamela, Jalisco Mexico. Size class I (DBH < 10 cm), Size class II (10 cm <DBH < 20 cm), Size class III (DBH > 20 cm). This map was created in Quantum GIS v.3.4 (Quantum GIS Development Team (2018). Quantum GIS Geographic Information System. Open Source Geospatial Foundation Project. http://qgis.osgeo.org).

**Figure 2:**
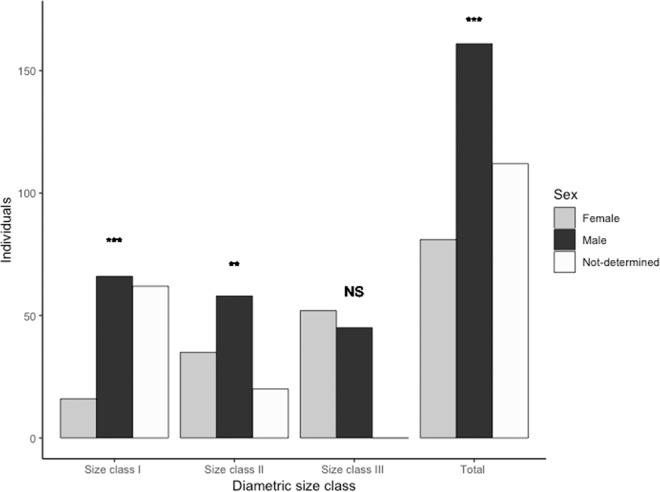
Number of individuals in each size class for each sex and for not-determined individuals over two reproductive seasons of *Spondias purpurea* within a 30 ha plot in Chamela Jalisco, Mexico. Notes: ***indicates p < 0.001, **p < 0.05, NS: p > 0.05 (G-test goodness of fit).

A total of 354 *S. purpurea* individuals were mapped within the 30 ha plot, 144 individuals were included in size class I, 113 in size class II and 97 individuals in size class III (Fig. 1). During two reproductive seasons we observed 62 size class I and 32 size class II individuals that did not flower, these individuals were included as non-reproductive individuals (Fig. 2). We observed significant differences in sex ratios across size classes (G _heterogeneity_ = 22.809, d.f. = 2, p = 0.0011). A male-biased sex ratio was observed for the pooled population and for size classes I and II (p < 0.05; Fig. 2). For size class III, the sex ratio did not significantly deviate from unity (p > 0.05) (Fig. 2). We observed significant differences in diameter size distribution between sexes (Fig. 2, D = 0.33, p < 0.001).

### Genetic diversity analysis

All 354 mapped individuals were genetically analysed. All nine loci were polymorphic and had similar levels of genetic diversity across size classes (Table 1). For the entire population, the average number of alleles per locus was N_a_ = 6.51 (±0.231), the allelic richness was A_r_ = 6.07 (±0.582), the observed and expected heterozygosities were H_o_ = 0.430 (±0.09) and H_e_ = 0.502 (±0.006) respectively.

**Table 1:** Genetic diversity estimates for each size class within 30 ha plot of *Spondias purpurea*.

Size class	N	N_a_	***A***_***r***_	H_o_	H_e_	F
Size class I	144	6.333 (0.273)	6.02 (1.05)	0.41822 (0.077)	0.49947 (0.078)	0.116 (−0.0692–0.3636)
Size class II	113	6.444 (0.297)	6.08 (1.01)	0.43545 (0.071)	0.49751 (0.075)	0.072 (−0.1256–0.3179)
Size class III	97	6.778 (0.037)	6.12 (1.08)	0.43775 (0.082)	0.51023 (0.078)	0.099 (−0.1151–0.3545)

N: Number of individuals; N_a_: mean allele number per locus (±SD); A_r_: Allelic richness (±SD); H_O_: observed heterozygosity (±SD); H_e_: expected heterozygosity (±SD); F: fixation index (Bootstrap estimates of confidence intervals).

#### Fine scale genetic structure and spatial analysis

**Figure 3:**
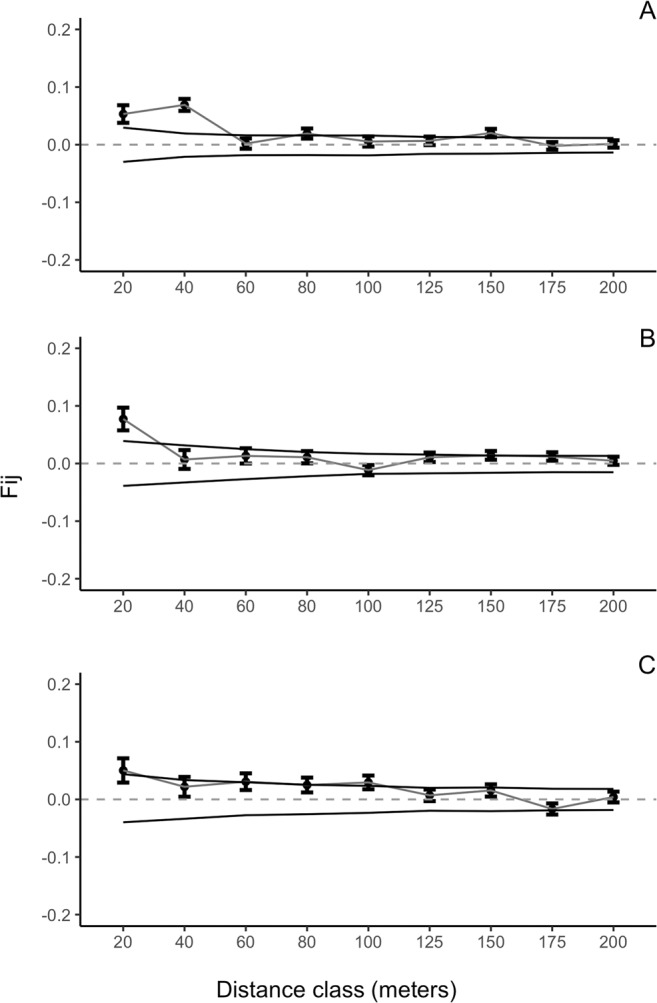
Autocorrelograms for estimated pairwise kinship correlation *F*_*ij*_ for three diametric size classes: (**A**) size class I (DBH < 10 cm), (**B**) size class II (10 <DBH ≤ 20 cm), (**C**) size class II (DBH > 20 cm). Each point represents calculated r_j_ values for each distance class. The solid lines represent upper and lower 95% confidence envelopes for the null hypothesis of no correlation (r_j_ = 0).

**Figure 4:**
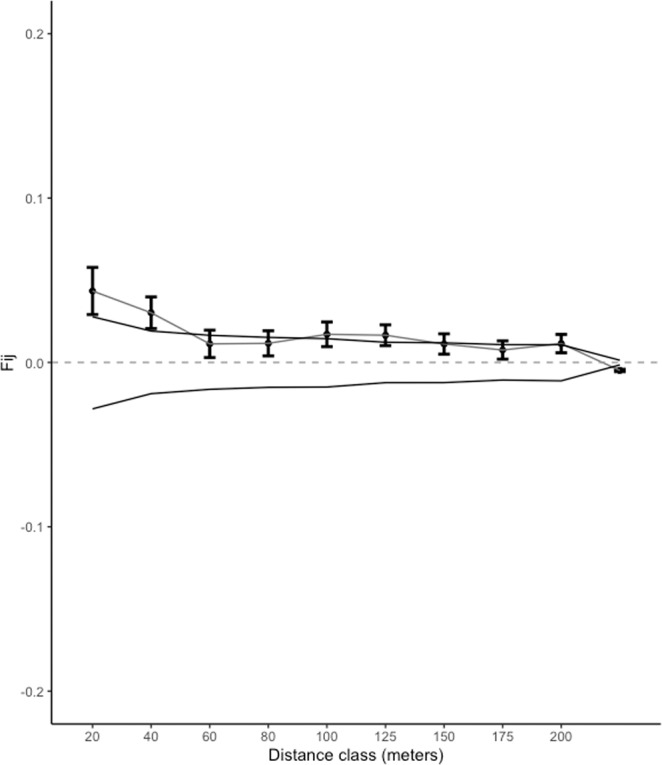
Kinship values (F_ij_) of size class I - size class III pairs. Each point represents calculated r_j_ values for each distance class. The solid lines represent upper and lower 95% confidence envelopes for the null hypothesis of no correlation (r_j_ = 0).

**Figure 5:**
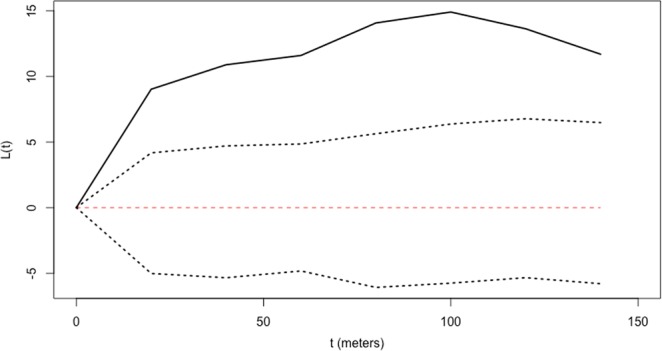
Results of Ripley ´s bivariate L(t) analyses for size class I and size class III individuals in a 30 ha plot located in Chamela, Jalisco, Mexico. Solid lines represent the L(t) values. Dotted lines represent 95% confidence intervals. Spatial association between size classes is suggested if values of L(t)>0. Conversely, L(t) <0 indicates repulsion between size classes. If L(t) values fall within the confidence envelope, the spatial relationship between size classes is deemed random.

Our results indicated relatively low levels of FSGS across different size classes. Individuals of size class I had significant but low pairwise kinship coefficient for the 20 and 40 m distance classes, F_ij_ = 0.0531 an F_ij_ = 0.069 respectively (Fig. 3A). Individuals of size class II had significant FSGS for the 20 m distance class (F_ij_ = 0.077) (Fig. 3B), similarly size class III individuals showed significant family structure for the 20 m distance class (F_ij_ = 0.05) (Fig. 3C). Although significant for the first distance classes, kinship coefficients were low (F_ij_ ≤ 0.077) in this *S. purpurea* population. The *Sp* statistic confirms these results, with all regression slopes being statistically significant (Table 2). The between-size class FSGS analysis, showed that size class I individuals exhibited low genetic relatedness with nearby size class III individuals (Fij = 0.043 and Fij = 0.030 for 20 and 40 m respectively), than expected for parent-progeny pairs (Fij $\simeq$ 0.5) (Fig. 4). Results of spatial interaction analyses indicated spatial association between individuals of size class I and III (Fig. 5).

**Table 2:** Slope of the regression (*b*_*log*_) between kinship coefficient and the log of the geographic distance between individuals, the average kinship between individuals separated by less than 20 meters (F _(1)_) and the *Sp* statistic calculated for each size class within the plot.

Size class	b_log_	F_(1)_	Sp
Size class I	−0.0121^NS^	0.0531	0.0128
Size class II	−0.01^NS^	0.0771	0.0108
Size class III	−0.01^NS^	0.0502	0.0105

NS: Not significant.

#### Parentage analysis

**Figure 6:**
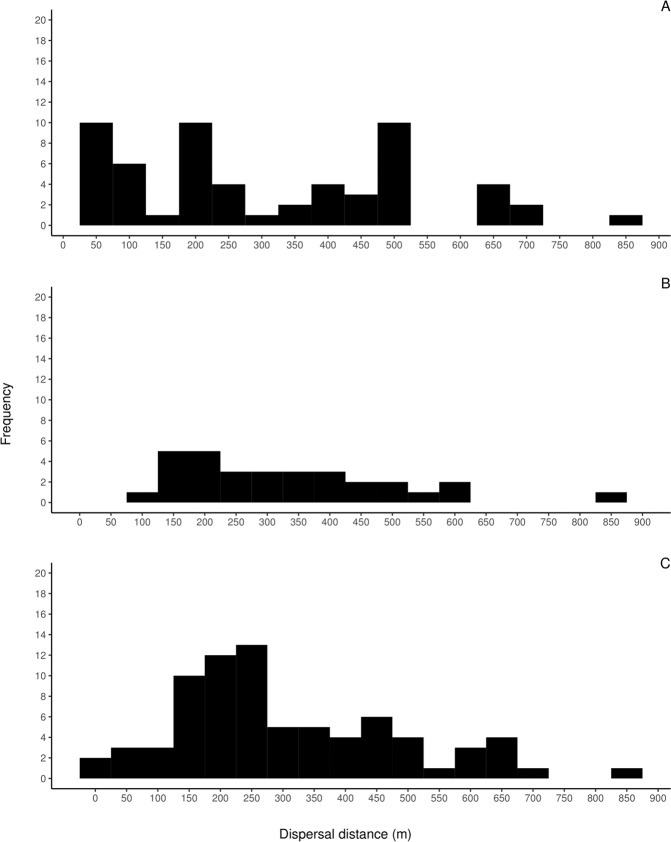
Frequency distribution of dispersal event distances: (**A**) Effective pollen dispersal, (**B**) Realized pollen dispersal and (**C**) Realized seed dispersal. Pollen dispersal is based on the results of a paternity assignment on seeds (n = 127) collected from 10 *Spondias purpurea* trees. Effective pollen and seed dispersal is based on parent-pair assignment on *Spondias purpurea* seedlings in the diametric size class I (n = 144) within of 30 ha plot in Chamela.

The exclusion probability estimated over all individuals and over nine loci was 99.99***%****.* Out of 127 genotyped seeds for effective pollen dispersal estimates, 68 were assigned to pollen donors within the plot, 58 of which with 95% confidence. We were able to identify six pollen donors within the plot that sired between 5 and 12 seeds (mean = 9.6 seeds by pollen donor). Effective pollen dispersal distances ranged between 27.9 m and 828.2 m with an average of 306.78 m (±28.57) and a median of 263.29 m. Approximately 30% of all effective pollination events assigned through paternity analysis, occurred between trees that were less than 150 m apart, 70% occurred between trees >200 and 600 m apart and 12% occurred between trees 600 to 850 m apart (Fig. 6A).

We determined realized pollen dispersal distances on 144 individuals in size class I, 31 were assigned to a parent-pair within the plot, 29 of which were assigned with >95% confidence. We were able to identify 11 pollen donors within the plot that sired between 1 and 5 established seedlings by each donor (mean = 2.8 seedling by pollen donor). The realized pollen dispersal distance ranged from 96.6 m to 854.6 m with an average of 331.6 m (±31.14) and a median of 293 m. For realized pollen dispersal, only 6.4% of the events occurred between trees <150 m apart, 93% occurred between trees >150 m apart and 12% occurred between trees 600 to 854 m apart (Fig. 6B).

The same 144 individuals of the previous analyses were used to estimate seed dispersal distances, 77 of these seedlings were assigned to a mother (13 maternal plants) within the plot (67 with >95% confidence). Seed dispersal distances ranged between 7.2 m and 833.2 m with an average of 305 m (±20.6) and a median of 255.9 m, 19.4% of all seed dispersal events occurred at distances <150 m apart from assigned maternal trees, 80.5% occurred between 150 m and 600, and 12.9% occurred between 600 and 833 m apart from the assigned maternal trees (Fig. 6C).

We did not find any significant evidence of a relationship between the number of established progeny produced by an individual and its DBH (r^2^ < 0.44, p > 0.144), nor between the number of progeny established and their distance from any given maternal tree (r 2 = 0.09, p = 0.99).

## Discussion

Sex ratios in the studied *S. purpurea* population were significantly male-biased, but departures from 1:1 ratio varied across sizes classes. While smaller size classes (DBH < 20 cm) were mostly male-biased, the largest size classes (>20 cm) did not deviate from a 1:1 sex ratio. In the two sampling periods of this study, we did not find differences in flowering frequency between male and female *Spondias* trees across size classes. Differences in size distributions between males and females are unlikely due to greater growth rates in males, but instead by an overrepresentation of males in the smaller size classes. The 1:1 sex ratio in the larger size classes probably indicates that many of the non-flowering individuals in the smaller size classes are likely females. Thus, *S. purpurea* females reach age at first reproduction at a minimum threshold size determined by a minimum budget of resources necessary for flower and fruit production. Flower production in males start at an early stage in individuals that are 2 cm in diameter and less than 1 m in height; while in females, flowering starts when they grow taller than 5.2 cm in diameter and >1 m in height. This result suggests precocious male reproduction in S. purpurea, as also found in other tropical species^36–41^. Female trees likely require a minimum branch size to bear fruit (i.e. >1 cm) which is related to the overall development of the plant.

We observed intermediate levels of genetic diversity in all three size classes of *S. purpurea* as expected for tropical trees^42–44^ and for dioecious species with obligate outcrossing^45,46^. Our data showed significant but low fine-scale structure up to 40 meters for the smallest size class and up to 20 meters for size classes II and III. *Sp* values were comparable to estimates for animal dispersed tropical trees (Sp ∈ [0.01…0.03]) and showed no evidence of FSGS across all size classes, which indicates that genotypes were largely randomly distributed in space. These results contradict expectations of high and significant structure in the smaller size classes of animal dispersed trees due to limited seed dispersal^20,45,47,48^, and a reduction in FSGS in larger diametric size classes due to post-dispersal demographic thinning (e.g. density-dependent predation and competition)^20,45,48–50^. Our expectations were based on previous findings for other vertebrate dispersed tropical dioecious tree species such as *Pouteria reticulata* (Sapotaceae), *Simarouba amara* (Simaroubaceae), *Ficus cyrtophylla* (Moraceae), and *Protium spruceanum* (Burseraceae); where significant FSGS was detected in sapling and adult size classes at short distances, and FSGS declined in larger diametric classes^20,45,51,52^. This pattern has also been shown for dioecious species such as *F. hispida, F. exasperata, F. pumila, Ceratiola ericoides, Eurya emarginata, Myracrodruon urundeuva*^46,53–56^; however this is not the pattern that we found for *S. purpurea* at Chamela, our data shows low genetic structure and no differences among size classes.

The unexpected low FSGS in *S. purpurea* is likely a result of long-distance seed dispersal and early density-dependent factors shaping FSGS patterns. In the study area two bird species (*Ortalis poliocephala* and *Icterus pustulatus*), eight mammal species (*Odocoileus virginianus*, *Pecari tajacu*, *Canis latrans*, *Nasua narica*, *Urocyon cinereoargenteus*, *Didelphis virginiana*, *Sciurus colliaei* and *Liomys pictus*) and one reptile (*Ctenosaura pectinata*) have been documented to feed on *S. purpurea* seeds^57^. Collared peccaries (*P. tajacu)* predate seeds^57^, however the rest are all potential dispersers that are capable of long-distance seed dispersal. Only three species consumed and left seeds underneath tree crowns (i.e. *D. virginiana*, *S. colliaei* and *I. pustulatus*), while the other species moved fruits away from maternal trees^57^. Intact seeds were observed in feces of *C. latrans*, *N. narica*, *U. cinereoargenteus* and *C. pectinata*; regurgitated seeds were also observed on rest spots and roosting sites of *O. virginianus* and *O. poliocephala*, respectively^57^. Seed dispersal studies assume that most seed dispersal follows a leptokurtic distribution, in which most seeds are dispersed nearby maternal trees^58^. However, seed dispersers may collect seeds on one tree and move them to other conspecifics in their feeding routes^59–62^. For most animal dispersed plant species, ingested seeds are normally dispersed away from maternal trees^62–64^. This seed dispersal behaviour has two consequences on gene flow patterns associated with seed dispersal. First, it promotes gene movement over long distances. In our parentage analysis, we were able to assign maternity to 53% of offspring within the plot, 85% of these assignments occurred at distances over 150 m away from the maternal tree. We deduced that offspring not assigned to maternal trees within the plot may be the result of seed dispersal events by mothers located outside our plot. Our results are comparable to paternity assignments obtained for other species using microsatellite markers^10,65,66^. Seed dispersal distance is an important result, as most studies that analyze seed dispersal rarely estimate seed dispersal over such long distances^9,20,67,68^. Second, dispersers may consume seeds from multiple trees before depositing them in resting places, creating a mixture of different genotypes^64^; both factors should reduce FSGS. Similar to seed dispersal, effective and realized pollen flow occurred more frequently at distances greater than 150 meters (70% and 93% respectively), suggesting that for *S. purpurea* insects also provide long-distance gene dispersal services, comparable to other tropical trees^69^.

In addition to long-dispersal gene flow, at shorter distances other factors may operate to limit FSGS and male and female fitness. Spatial association results between the smallest size class and the largest size class indicated that there is recruitment near conspecific trees, however only if the genetic relatedness between these two size classes is low (Figs. 4 and 5), suggesting a parentage recruitment effect. Furthermore, based on pre-dispersed seeds (effective pollen dispersal) paternity analysis show that 27% of pollen donors are located within 100 meters of maternal trees (Fig. 6A), meanwhile parent-pair analyses of established seedlings (realized gene flow) showed that less than 7% of all seedlings had a parent within the same distance range (100 m) (Fig. 6B,C). In other words, seeds sired by distant male trees have a higher likelihood of establishing than seeds sired by nearby males; in the same way seeds dispersed away from maternal trees have a greater probability of germination and survivorship in comparison with seed dispersed at shorter distances. This indicates that the probability of surviving and establishing in the population is a function of distance to their parental trees (both male and female) congruent with the Janzen-Connell (J-C) hypothesis^13,14^. According to J-C, recruitment is limited in nearby neighborhoods of paternal plants due to asymmetric competition and predator and pathogen transferal from related nearby individuals. Thus, dispersal away from parental genotypes should increase the survival probability of seedlings, thereby in accordance with our paternity data, lower FSGS could be indicative of strong J-C effects. In our study site, Mendez-Toribio and colleagues^70^ compared recruited seedling richness and density underneath female and male *S. purpurea* trees. They found that seedling density and richness was higher underneath female trees compared to male trees; furthermore density of zoochorous species was greater underneath the canopy of *S. purpurea* females suggesting a directional dispersal bias towards them, mediated by seed dispersers^70^. Interestingly no seedlings of *S. purpurea* were recorded directly underneath conspecific trees possibly due to strong negative density dependent effects (*e.g*. seed predation, interspecific competition)^70–72^ reinforcing the idea that J-C effects strongly shape *S. purpurea* seedling recruitment.

To our knowledge only two studies have previously shown J-C effects using genetic data, in *Pinus halepensis* (Pinaceae), Steinitz and colleagues^73^ used parentage analysis to estimate differences among seed dispersal kernels and effective dispersal kernels demonstrating higher seedling survival with increasing distance from maternal plants. Similar results were found by Berens and colleagues^74^ in the vertebrate seed dispersed tropical tree *Prunus africana* (Rosaceae); by comparing dispersal distances among different seedling stages, they found that dispersal distances were higher in older seedlings.

Our paternity analyses also showed evidence of other factors that affected individual plant fitness in *S. purpurea* including pollen and seed dispersal distances. Previous studies have analysed individual plant fitness associating only fecundity (seed production) with tree size indicating that in several species, the size of the plant is positively related to greater fecundity^75^; however, information on the success of these seeds to survive and establish in the population is scarce^75^. Genetic markers are a useful tool to trace the number of offspring recruited in the population sired or dispersed by a particular parental tree, data that can be used as an individual fitness measure^75^. Our results indicated that there is not a relationship between the number of established progeny and tree size (DBH); larger individuals could have the potential to produce more offspring due to higher fecundity^75^, however, other factors can also influence progeny success (e.g., timing of germination, seed predation, dispersal of offspring). In addition, the number of progeny assigned is not a linear function of the distance from its maternal tree. Our realized seed dispersal data suggests a population recruitment curve (PRC) between 150 and 250 meters away from the maternal tree (Fig. 6C). In accordance with the original proposal of Janzen^13^ PRC is the result of the interaction between seed dispersal and seed or seedling predation^13^. In addition, our results show that the effective pollen flow distances are also comparable to the realized seed dispersal PRC. The proposal by Janzen^13^ did not originally considered pollen flow in the model; interestingly, our results show that Density Dependent (DD) factors also regulate effective pollen dispersal distances (Fig. 6B). Therefore, DD factors may regulate the parental structure (both maternal and paternal) of the established progeny, also regulating the spatial distribution of genetic diversity within populations.

By comparing effective and realized pollen dispersal results, we can observe that seeds sired by males separated by at least 150 m from female trees have a greater likelihood of establishing in the population than seeds sired by nearby male trees. As shown for several high density insect pollinated tropical trees, effective pollination with nearby neighbours also occurred in *S. purpurea*^76,77^. However, our realized pollen flow data showed that 85% of established seedlings are sired by pollen donors that are >150 m away from maternal trees, indicating that long distance sires have higher male fitness. Similarly, our realized seed dispersal data revealed that seedlings are dispersed more frequently at distances over 150 m, indicating that long dispersal distances from maternal trees also had a strong effect on individual seedling fitness. It has been proposed that in order to coexist with hermaphrodites, dioecious species could have compensatory fitness mechanisms^58,78^ for example in some tropical forests females of dioecious species produce more seeds than hermaphroditic species^79^. However, such greater seed production can result in greater seed deposition nearby maternal female trees due to limited seed dispersal; consequently, more aggregated seedlings should be expected and this aggregation could lead to a reduction in fitness via decreased seedling performance^15,80,81^. Under this scenario dioecious plants may allocate more resources to increase seed dispersal to enhance fitness as we show here for *S. purpurea*.

In summary, we evaluated the population sex ratio and the effects of gene dispersal through effective and realized seed and pollen dispersal on FSGS across size classes of the dioecious tree *S. purpurea*. Our results indicated that the male biased sex ratio of this species is due to precocious male reproduction; and the low levels of FSGS among size classes is likely determined by a combination of factors related with long dispersal gene flow and J-C effects during early recruitment stages. J-C density dependent factors regulate realized gene flow patterns and fitness in *S. purpurea*; both long-distance pollen donors and long-distance seed dispersers have higher male and female fitness, respectively, all of which result in low FSGS. Therefore, we propose that low levels of FSGS in tropical trees may be indicative of density dependent factors regulating seedling populations of tropical trees.

## Materials and Methods

### Study area and study species

This study was conducted in the Chamela-Cuixmala Biosphere Reserve at Chamela Biological Station, UNAM (ChaBS) (19°30′N, 105°03′W) in the central Pacific coast of Jalisco, Mexico. This reserve protects 13,142 hectares of Tropical Dry Forest (TDF), a highly endangered ecosystem. Permission for sampling was obtained from ChaBS director.

*Spondias purpurea* L. (ANACARDIACEAE) is a small (3–10 m), deciduous and dioecious tree native to the tropical dry forests of Mexico and Central America^82,83^. Reproduction occurs between December and May. This tree depends on insects for cross pollination, primarily stingless bees and wasps^84^. Female trees produce small, bright red, juicy and sweet-acidic fruits that are eaten by various mammals and birds^57^. These fruits represent an important source of water and nutrients in the dry season when high temperatures, water and food scarcity are commonplace^57,70^.

### Experimental design and sample collection

We set a 30 ha plot (1000 × 300 m) where all *S. purpurea* individuals were tagged and geo-referenced. Plants were grouped into size classes using diameter at breast height (DBH), except for trees less than 2 m tall, for which we used the diameter at the base of the stem. We defined three size classes: class I (DBH < 10 cm), class II (10 cm <DBH < 20 cm), class III (DBH > 20 cm). Seedlings were included in size class I. Sexual expression was monitored for all individuals in two consecutive flowering seasons (2015, 2016). We collected flowers from different sections of the canopy to accurately determine the gender of all individuals.

To estimate pollen dispersal distances we collected twenty fruits from ten randomly chosen female trees within the plot. Fruits were collected directly from the canopy of each individual to guarantee that we sampled progeny from a single maternal tree.

### Sex ratios

Sex ratio was expressed as the proportion of males in the population: males/ (females + males)^85^. For each sex we determined the frequency of individuals in each diametric size class. To determine deviations from 1:1 sex ratio we performed a goodness of fit G-test for each size class and for pooled size classes. To evaluate differences in diameter size distribution between sexes, we used a Kolmogorov-Smirnov two sample test. Statistical analyses were performed using the *RVAideMemoire*^86^ and *stats* libraries implemented in the R computing environment^87^.

### DNA extraction and microsatellite amplification

We collected fresh leaf tissue from all individuals within the 30 ha plot and stored them at −20 °C until DNA extraction. For pollen dispersal analyses we dissected fruits and sampled embryos for DNA extraction. DNA from leaves and embryos was extracted using a modified Cetylmethylammonium Bromide (CTAB) protocol^88^. Nine microsatellites previously developed for *S. purpurea*^89^ were amplified via multiplex PCR using QIAGEN Multiplex kit (QIAGEN, Hilden, Germany) in 12 µL reaction volumes. The first multiplex mix used primers SPUR44, SPUR40, SPUR28 and SPUR41, the second mix included the primers SPUR35, SPUR29 and SPUR33 and the third set contained the primers SPUR42 and SPUR39; in all cases primers were at a 0.2 µM concentration. The PCR amplification profile included an initial activation step of 95 °C for 15 min, followed by a touchdown PCR consisting of 32 cycles with denaturation at 95 °C for 90 s; annealing for 60 s with temperature decreasing 1 °C every two cycles from 64 °C (12 cycles), then 10 cycles at 58 °C and 10 cycles at 57 °C; elongation at 72 °C for 60 s; and a final extension at 72 °C for 5 min. Fragments were analysed on an automatic ABI-PRISM 3100-Avant sequencer (APPLIED BIOSYSTEMS, Carlsbad, California, USA) using GeneScan LIZ 600 (APPLIED BIOSYSTEMS, Carlsbad, California, USA) to determine fragment sizes. Alleles were scored manually using *GeneMarker Software* version 2.6.4 (SOFTGENETICS). To reduce genotyping error, all samples were independently genotyped by two different researchers to reach consensus in the final data set. The primer SPUR57 was excluded from the analyses because in several individuals it produced multiple peaks that could not be genotyped properly. Erroneous genotypes may critically bias paternity assignments and eliminating SPUR57 did not significantly reduce the exclusion probability of the remaining 9 markers.

### Genetic diversity analysis

Genetic diversity was quantified for each size class using the following parameters: allele number averaged across loci (*Na*), Allelic richness (*Ar*), observed (*Ho*) and expected heterozygosities (*He*) and fixation indexes (*F*). These parameters were calculated using the software *ARLEQUIN*^90^. Confidence intervals for *F* values were obtained by bootstrapping over loci using the library *hierfstat*^91^ implemented in R^87^.

### Fine scale genetic structure and spatial analysis

Fine-scale genetic structure was estimated for all size classes using the software *SPAGeDi*^92^. We estimated the mean and the confidence intervals of pairwise kinship coefficients between all pairs of individuals within each size class. For each analysis, we selected eight distance categories; the first five categories are separated by 20 m intervals up to 100 m, with the remaining categories separated by 25 m intervals up to 200 m. All categories included more than 100 pairwise comparisons. To test the null hypothesis of a random distribution of genotypes in space, estimated *F*_*ij*_ values were compared to the 95% confidence intervals generated by 10,000 random permutations of individuals in space. *F*_*ij*_ values were regressed onto ln (*d*_*ij*_), where *d*_*ij*_ is the spatial distance between all pairs of *ij-*individuals. To quantify the FSGS intensity and to compare it with other studies the *Sp* statistic was calculated according to Vekemans and Hardy^3^. The significance of the slope was assessed by comparing the estimated value with 10 000 random shifts of individuals among locations. Standard errors (SE) for all slopes were determined by jackknifing across loci. In order to analyze parentage and conspecific effects on seedling recruitment, we tested the spatial interaction (i.e., association or repulsion) and inter-size class genetic structure between the smallest size class (size class I) and the largest size class (size class III). To evaluate relatedness patterns among plants between size class I and III we performed a “between-size class” FSGS analysis; in which relatedness is calculated for pairs of individuals including an individual from size class I and an individual from size class III, yielding the degree of relatedness of each “seedling” with their closest adults. The distances classes analysed and parameters used for to test the null hypothesis of a random distribution of genotypes in space were the same used in the FSGS within size classes. The spatial interaction (association or repulsion) between individuals of size class I and III was evaluated using Ripley’s second order K_12_(t) function. K_12_(t) estimates the expected number of type 1 (size class I individuals in this study) within a distance (t) of a randomly chosen type 2 point (size class III). To reduce scale dependencies and stabilize variances, we used the L (t) square root transformation of K (t)^21,93^. Spatial interaction was evaluated for t distances between 0 and 150 m and an isotropic edge correction was also applied. We chose ‘random labeling’ as the null hypothesis to construct confidence envelopes^94^. Confidence envelopes were constructed from 999 permutations. Spatial association between size class I and size class III individuals is suggested if values of L(t) >0. Conversely, L (t) <0 indicates repulsion between size class I and size class III individuals. If L(t) values fall within the confidence envelope, the spatial relationship is deemed random. Spatial analysis was conducted using the spatstat library^95^ implemented in the R computing environment^87^.

### Parentage analysis

To determine gene flow levels via pollen and seeds we performed two separate parentage analysis. We first evaluated effective pollen dispersal via paternity analysis on seed embryos as offspring genotypes. Males that reproduced in the same year when seeds were collected were considered potential fathers in the analysis. The effective pollen dispersal distance was calculated as the distance between the father assigned to the embryo and the maternal tree. In a second analysis we evaluated realized pollen and seed dispersal distances. To this end we used the genotypes of individuals in the diametric class I as offspring, while reproductive individuals (i.e. identified as male or females by flower observation) in diametric classes II and III were used as potential parents. These analyses resulted in two dispersal distances: (i) the realized pollen dispersal distance estimated as the distance between the mother and father assigned to an established seedling when both parents occur within the plot^20^; (ii) and the effective seed dispersal distance calculated as the distance between seedlings and their assigned mother. Parentage analyses were conducted using Colony V2^96^. We used the parameter of a polygamous outcrossing dioecious species, and selected the full likelihood method with a large run. We assigned paternity using “strict” (>80%) confidence levels. We included a genotyping error probability of 0.0923 based on the number of mismatches observed between the genotypes of maternal trees and their offspring (calculated from the data set of embryos with known maternal genotypes), estimated using Cervus 3.0^97^.

## Data Availability

The datasets generated and analysed during the current study are available from the corresponding author upon request.
